# Cerium Binding Activity of Pectins Isolated from the Seagrasses *Zostera marina* and *Phyllospadix iwatensis*

**DOI:** 10.3390/md10040834

**Published:** 2012-04-05

**Authors:** Yuri Khotimchenko, Elena Khozhaenko, Valeri Kovalev, Maxim Khotimchenko

**Affiliations:** 1 School of Biomedicine, Far Eastern Federal University, 8, Sukhanova str., Vladivostok, 690091, Russia; Email: varitas@live.ru (E.K.); maxkhot@yandex.ru (M.K.); 2 A.V. Zhirmunski Institute of Marine Biology, Far Eastern Branch of Russian Academy of Sciences, 17, Palchevskgo str., Vladivostok, 690059, Russia; Email: vostokpharm@mail.ru; 3 Vostokpharm Co., LTD., 17, Palchevskgo str., Vladivostok, 690059, Russia

**Keywords:** cerium, equilibrium study, pectin, degree of esterification, removal

## Abstract

Cerium binding activity of three different water soluble pectin compounds of different origin was studied in a batch sorption system. The Langmuir, Freundlich and BET sorption models were adopted to describe the binding reactions between metal ions and pectin molecules. The Langmuir model provided the best fit. Within the pH range from 4.0 to 6.0, the largest amount of the cerium ions was bound by pectin isolated from the seagrass *Phylospadix iwatensis* in comparison to pectin extracted from the seagrass *Zostera marina* and pectin obtained from citrus peel (commercial grade). The Langmuir constants were also highest for the pectin samples isolated from the seagrass *P. iwatensis*. The results obtained from this study suggest that pectin is a prospective source for the development of radioisotope-removing pharmaceuticals.

## 1. Introduction

Pectin substances are considered as an essential primary cell wall component of all higher flowering plants. The average contents of pectins in the cell walls may achieve 30%. Along the other parts, pectins provide durability and expansibility of the cell walls as well as maintain numerous functions necessary for the growth and development processes of plants, and further determine resistance of plants against diseases [1]. Pectins are industrially extracted from pectin-rich sugar-beet pulp, apple pomace and citrus peels [2] and commonly used in food industry as gel-forming, thickening and stabilizing agents and emulsifiers. Besides the ground plants, pectins can also be obtained from marine plants such as seagrasses belonging to the family Zosteraceae [3]. Areas where seagrasses belonging to the Zosteraceae family grow include seas in the northern and southern hemispheres where the climate is moderate. Also several species are found in tropical seas. *Phyllospadix* grows in the coastal waters of the Sea of Japan, the Sea of Okhotsk and near the Pacific coast of North America, whereas *Heterozostera* is located in the coastal waters of Australia and Chile. Seagrasses are generally found in littoral and sublittoral zones in depths not exceeding 21 m. They form dense tangles in large masses. Oriental seas near the Russian coastal line form an area for the growth of three species of *Zosteras*. The most common of them are *Zosteras marina* and *Zosteras asiatica*. The third specie, *Zosteras nana* is quite rare and does not form considerable plant concentrations. Reproduction of the marine *Zostera* usually happens through two ways: vegetative and reproductive; the former is considered to be more effective. *Z. marina *usually grows in the coastal zone with the depth varying from 0.3 to 11 m, in particular, from 1 to 5 m. Width of the tangles varies from 50 to 600 m and the weight, from 0.3 to 12 kg/m^2^. Resources, in Peter the Great Bay of the Sea of Japan alone, are estimated to be 16,000 tons, wet weight. Supplies of *Z. asiatica* are also considerable. Quantities in the coastal areas of Primorskiy Region in Russia amount to about 28,000 tons. The total weight of *Phyllospadix iwatensis *growing on the continental coast of Japan Sea is about 140–190 thousand tons [3]. Therefore, considerable stocks of seagrass in the Russian Far East seas present a background for creation of industrial technologies of marine raw materials manufactured for the needs of the pharmaceutical industry. It should be mentioned that pectins of marine origin have not yet been used in the food industry.

A general pectin model structure has been proposed by Perez and collaborators [4]. Structure of pectins is quite variable in different plants and cell types due to the differences in the polymer size, acetylation type, degree of esterification and other variances of the galacturonic acid in the main homogalacturonan (HG) chain and variations in the side chain length and type of rhamnogalacturonan-I (RG-I). It was found that pectins contained in seagrasses, in particular, in Zosteraceae in comparison with the plant pectins, are characterized by slight structural differences such as presence of arabinogalactan I (AG-I) and AG-II and some other types of sugars [5,6,7]. Depending on the degree of methyl esterification (DM), which is defined as the percentage of galacturonic acid (GalA) residues esterified with methanol, pectins are referred to as high-methoxyl pectins (HMP) (DM ≥ 50) or low-methoxyl pectins (LMP ) (DM < 50). Commercial LMPs are manufactured from HMPs by acid, alkali, ammonia, or enzymatic de-esterification. The main raw materials, which are used to produce commercial LM and HM pectins, are apple pomace and citrus peels [8]. These industrial byproducts are also sources of dietary fibers [9,10]. Other dietary fiber-rich (as well as pectin-rich) sources such as seagrasses have also been reported [11]. However to date, pectin substances extracted from seagrasses have barely been studied.

Nowadays, pectins are an important nutrient in the human diet as they are a major component of dietary fiber and have been reported to bind heavy metals, to lower serum cholesterol levels and to have immune-stimulating and anti-ulcer activities [12,13]. Due to their anion character, pectins are used as cation exchangers for the removal of metal cations from aqueous solutions [14,15]. There are also some results confirming the possibility for application of the pectins for removal of heavy metals from human body [16]. Moreover, the efficacy of the apple pectin in reduction of ^137^Cs-storage in the “Chernobyl” children body was recently shown [17].

The ability of pectins to bind cations is caused by the presence of non-methyl esterified galacturonosyl residues. LMPs form gel in the presence of calcium due to the formation of ionic cross-links between homogalacturonan chains. The mechanism of gelation is not fully understood although one model called “egg-box” has received much attention [18]. In this model, calcium cross-linked junction zones are proposed to be formed between homogalacturonan chains containing at least six contiguous and non-esterified galacturonosyl residues. This results in the formation of a polymer network, in which water molecules are entrapped. The gelation of pectins and the physical properties of the gel are controlled by the extent of methyl esterification of the galacturonosyl residues and their interaction with divalent cations [18].

The goal of the present study was to compare the metal-binding activity of three types of pectins, including the sample isolated from the seagrass *Z. marina*, the sample isolated from the seagrass *P. iwatensis* and commercial grade pectin derived from citrus peels. The target element of the present study was cerium cations. Cerium belongs to the lanthanide series or rare earth elements exerting diverse biological effects mainly by their resemblance to calcium. Because of their diverse physical, chemical and biological effects, lanthanides have been used industrially in color TV, lasers, photographic cameras, semiconductors, binoculars, and movie films and also in medicine as anti-cancer, anti-inflammatory, and antiviral agents. Lanthanides-enriched fertilizers (mainly consisting of cerium, lanthanum, and neodymium nitrates) are known to be able to increase the yields of crops, but because plants can accumulate lanthanides, this group of biologically non-necessary elements therefore can be inevitably accumulated in the environment and then transferred to the human body through the food chain and might damage human body. Some *in vivo* studies showed that lanthanides could be accumulated in the liver, kidney, spleen, and lung, and had adverse effects on organs, e.g., a lesion caused by lanthanides showed oxidative stress, and disturbance of the homeostasis of essential elements and enzymes [19], thereby generating various inflammatory responses. They could promote enzymatic activities and the mRNA expression of cytokines during proinflammatory responses in mice and also could produce reactive oxygen in mice liver and caused spleen apoptosis [20,21]. Another important problem is the negative influence of radioactive cerium on humans. All in all, it is essential to find an effective and safe method to remove radioisotopes from the human body.

## 2. Results and Discussion

Determination of the physicochemical properties of the pectin samples showed that anhydrogalacturonic acid content in all samples was only slightly different, making up almost 80% of the pectin molecule. The remaining 20–30% of the pectin composition contains branched rhamnogalacturonan side chains made of such saccharides such as galactose, xylose, rhamnose and others, as was mentioned in a previous section. Amount of the free anhydrogalacturonic acid in the commercial citrus pectin sample was 2.2 and 1.9 fold lower than that in the pectins isolated from *Z. marina* and *P. iwatensis*, respectively. Conversely, intrinsic viscosity of the commercial pectin was 2.2 and 2.1 fold higher than that of the pectins from seagrasses, indicating the higher degree of polymerization of this pectin sample. Regarding the amount of the esterified carboxyl groups, the commercial citrus pectin was a typical high esterified pectin, with a degree of esterification higher than 50%. Pectin isolated from the seagrasses *Z. marina* and *P*. *iwatensis *were considered to be low esterified, with a degree of esterification of 7.9 and 10.2% respectively (Table 1). Therefore, the main differences between the three pectin samples studied were their rhamnogalacturonan composition, molecular weight and degree of esterification.

**Table 1:** Physicochemical characteristics of the pectin samples.

Pectin Sample	Total Anhydrogalacturonic Acid Content, %	Free Anhydrogalacturonic Acid Content, %	Intrinsic Viscosity, mL g^−1^ of Ahydrogalacturonic Acid	Degree of Esterification, %
Commercial citrus pectin	79.4	31.6	915	60.2%
Pectin from *Zostera marina*	71.5	68.4	415	7.9%
Pectin from *Phyllospadix iwatensis*	74.9	60.4	431	10.2%

**Figure 1:**
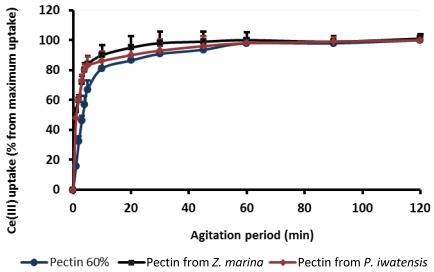
Cerium binding capacity of the three pectins as a function of the agitation period.

At the first stages of experiment, the optimal conditions providing interactive reactions between polysaccharide and the metal ions were estimated. Parameters of the binding kinetic were obtained estimating the amounts of the cerium ions bound by pectins over time, up to 120 min under stirring conditions. At this time, the maximum binding capacity was denoted as 100%. The plots displaying the relationship between pectin binding capacity and period of interaction show that 50% of the highest binding capacity was achieved in 15–20 min, whereas the saturation of the binding sites of the pectins occurred after approximately 60 min of interaction (Figure 1). For this reason, the following experiments focused on estimation of the cerium binding capacity of the pectins were carried out with intervals of interaction longer than 60 min.

**Figure 2:**
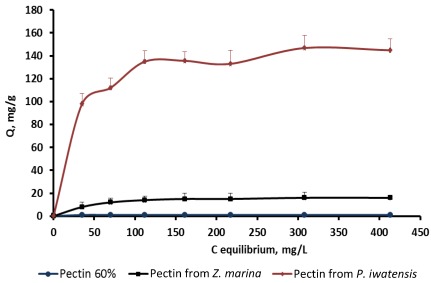
Isotherms of the cerium binding activity for the three pectin samples at pH 2.0.

On the basis of the quantitative data collected from the binding processes between the pectin samples and the stable cerium isotopes the equilibrium sorption isotherms were drawn (Figure 2–Figure 4). 

**Figure 3:**
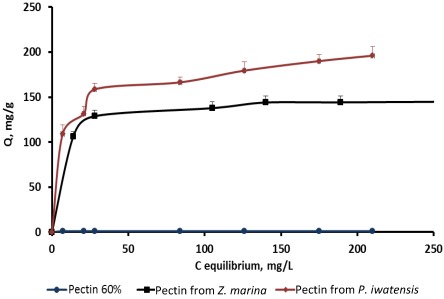
Isotherms of the cerium binding activity for the three pectin samples at pH 4.0.

**Figure 4:**
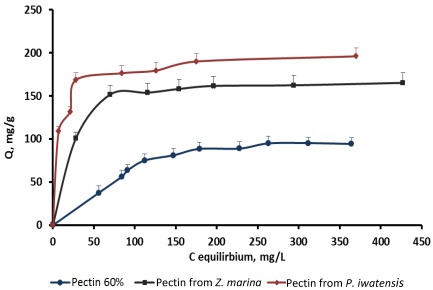
Isotherms of the cerium binding activity for the three pectin samples at pH 6.0.

It was confirmed that the experimental data exhibited a typical Langmuir isotherm shape. Therefore the Langmuir sorption model was used for the calculation of the maximum sorption capacity (q_max_) and affinity (b) coefficients according to the following equation, which is generally used to describe the equilibrium sorption isotherm for a monolayer sorption with a finite number of identical binding sites.

The Langmuir equation is more commonly used to describe equilibrium sorption isotherm, which is valid for monolayer sorption with a finite number of identical sites and is given by:


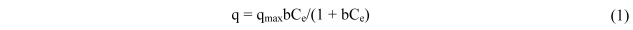


where q_max _is the maximum sorption at monolayer (mg g^−1^), C_e_ is a final equilibrium concentration of Ce^3+^, q is the amount of Ce^3+^ bound per weight unit of the pectin compound at final equilibrium concentration (mg g^−1^), b is the Langmuir constant related to the affinity of binding sites (mL mg^−1^) and is considered as a measure of the sorption energy.

Coefficient of the maximum sorption capacity is known to be the mathematical expression of the number of the active binding sites in the sorbent molecule, which can potentially interact and hold sorbate particles. Affinity constant indicates the stability of the bonds being formed during the binding reactions as well as velocity of these processes.

The following linearized plot of the Langmuir equation was used in this study:


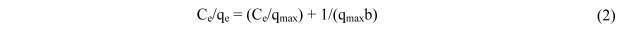


Taking into account that the form of the Langmuir isotherms is similar to the ones of Freundlich and BET models the constants of these models were also evaluated. The widely used empirical Freundlich equation based on sorption on a heterogeneous surface is given by:


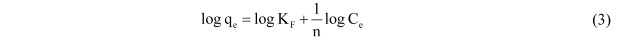


where K_F_ and n are Freundlich constants indicating sorption capacity (mg g^−^^1^) and intensity, respectively. K_F_ and n can be determined from linear plot of log q_e_ against log C_e_.

The BET equation is given by:


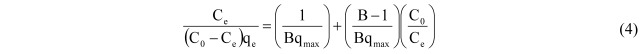


where q_max_ is the maximum uptake at monolayer (mg g^−1^), C_e_ is the equilibrium concentration of Ce^3+^ (mg L^−1^), C_0_ is the saturation concentration of the solute (mg L^−1^), q_e_ is the amount of Ce^3+^ bound per unit weight of the pectin compounds at equilibrium concentration (mg g^−1^) and B is the BET constant expressing the energy of interaction with surface.

Comparison of the sorption constants calculated with the use of the mathematical models mentioned above showed that highest cerium binding capacity is typical of the pectin isolated from *P. iwatensis*. At the pH 2.0 this was the only pectin showing a marked cerium binding activity (Table 2). At pH 4.0 the maximum sorption capacity constant of the pectin from *P. iwatensis* was 26.5% higher than that of pectin from *Z. marina* whereas commercial citrus pectin at this pH was not effective at all (Table 3). At the pH 6.0 the value of the maximum sorption capacity of the pectin isolated from *P. iwatensis *was higher than that of the pectin from *Z. marina* and commercial citrus pectin by 15.3% and 38.3% respectively (Table 4). Differences between affinity coefficients (b) of the different pectin samples regarding cerium ions were corresponding with those of maximum sorption capacity. Nevertheless, it should be noted that the affinity coefficients for both pectin samples isolated from seagrasses were very close and it was not possible to significantly mark the most pronounced one. At the same time the affinity coefficient of the commercial citrus pectin samples was markedly lower at pH 6.0, whereas at lower pH values it could not be calculated at all (-). The observed statistically signiﬁcant (at 95% conﬁdence level) linear relationships as evidenced by the *R*^2^-values (close to unity) indicate the applicability of the adsorption isotherms. Calculation of the Freundlich and BET constants showed that these models cannot be used for these pectin samples because their *R*^2^ coefficient was lower than 0.95 and the values obtained cannot be considered as significant.

**Table 2:** Langmuir, Freundlich and BET isotherm constants and correlation coefficients of Ce^3+^ binding capacity for different pectins at pH 2.0.

Sample	Langmuir		Freundlich				BET		
	B (mL mg^−1^)	q_max_ (mg g^−1^)	*R*^2^	K_F_ (mg g^−1^)	n	*R*^2^	Q (mg g^−1^)	B	*R*^2^
Commercial citrus pectin	-	-	-	-	-	-	-	-	-
Pectin from *Z. marina*	0.030	17.45	0.9982	1.744	3.778	0.8467	1.526	6.506	0.1512
Pectin from *P. iwatensis*	0.046	153.84	0.9976	5.816	6.207	0.8834	2.381	50.000	0.6959

**Table 3:** Langmuir, Freundlich and BET isotherm constants and correlation coefficients of Ce^3+^ binding capacity for different pectins at pH 4.0.

Sample	Langmuir		Freundlich				BET		
	B (mL mg^−1^)	q_max_ (mg g^−1^)	*R*^2^	K_F_ (mg g^−1^)	n	*R*^2^	Q (mg g^−1^)	B	*R*^2^
Commercial citrus pectin	-	-	-	-	-	-	-	-	-
Pectin from *Z. marina*	0.193	147.06	0.9999	7.016	10.834	0.8467	2.000	60.975	0.6739
Pectin from *P. iwatensis*	0.097	200.00	0.9967	6.803	6.157	0.9442	0.536	227.27	0.245

**Table 4:** Langmuir, Freundlich and BET isotherm constants and correlation coefficients of Ce^3+^ binding capacity for different pectins at pH 6.0.

Sample	Langmuir		Freundlich				BET			
	B (mL mg^−1^)	q_max_ (mg g^−1^)	*R*^2^	K_F_ (mg g^−1^)	n	*R*^2^		Q (mg g^−1^)	B	*R*^2^
Commercial citrus pectin	0.011	123.45	0.9695	2.461	2.253	0.8377		14.785	16.103	0.2039
Pectin from *Z. marina*	0.075	169.49	0.9994	6.191	6.105	0.7384		1.762	70.922	0.2959
Pectin from *P. iwatensis*	0.110	200.00	0.9993	7.027	6.891	0.8599		1.648	85.470	0.2666

The essential features of the Langmuir isotherm can be expressed in terms of a dimensionless constant separation factor, R_L _that is used to predict whether an adsorption system is “favorable” or “unfavorable”. The separation factor, R_L_ is defined by:


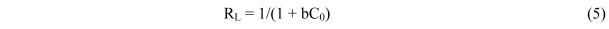


where C_0_ is the initial Ce^3+^ concentration (mg mL^−1^) and b is the Langmuir adsorption equilibrium constant (mL mg^−^^1^). The results of the R_L_ factor calculation (Table 5) showed that based on the effect of the separation factor on isotherm shape, the R_L_ values of all pectin samples were in the range of 0 *< *R_L _*< *1 indicating that the binding of Ce^3+^ by these substances is favorable.

The results suggest that pectin from *P. iwatensis *is the most suitable substrate for binding Ce^3+^ ions because the values of *b* and q_max_ for the other pectin compounds were lower, confirming the lower capacity of these substances to bind Ce^3+^. Although R_L _values of these pectin compounds were also between 0 and 1 and they also may be considered as favorable sorbents for Ce^3+^. It was found that the binding process is described by Langmuir model, which is valid for monolayer sorption with a ﬁnite number of identical sites. Therefore, one active site of the pectin molecule interacts is only one ion of metal. This corresponds to the “egg-box” model of the pectin-metal interaction according to which, the obvious mechanism of sorption is related to the formation of covalent and hydrogen bonds between the metal ions and non-esterified carboxyl groups and hydrogen atoms located on the pectin molecules and acting as the binding sites [18].

**Table 5:** R_L_ values of cerium binding by the different pectin samples based on the Langmuir equation.

**Ce^3+^ Initial Concentration,mg L^−1^**	**pH 2.0**			**pH 4.0**			**pH 6.0**		
	Commercial Citrus Pectin	Pectin from *Z. marina*	Pectin from *P. iwatensis*	Commercial Citrus Pectin	Pectin from *Z. marina*	Pectin from *P. iwatensis*	Commercial Citrus Pectin	Pectin from *Z. marina*	Pectin from *P. iwatensis*
20	-	0.620	0.517	-	0.206	0.340	0.819	0.397	0.312
40	-	0.450	0.349	-	0.115	0.205	0.694	0.248	0.185
60	-	0.353	0.203	-	0.079	0.146	0.602	0.180	0.131
80	-	0.290	0.211	-	0.061	0.114	0.532	0.141	0.102
100	-	0.246	0.176	-	0.049	0.093	0.476	0.116	0.083

**Figure 5:**
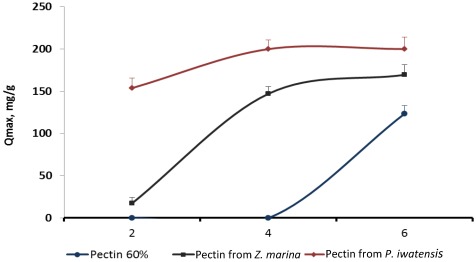
q_max_ values reflecting cerium binding capacity of the three different pectins at various pH levels.

Estimation of the possible influence of pH of the media on the binding capacity of the pectins showed that the largest amount of the cerium ions were bound by pectins being in the media with pH varying from 4.0 to 6.0. The increase of the pH higher than 6.0 resulted in formation of the insoluble Ce(OH)_4_ precipitate thus preventing binding processes. Cerium binding activity of the commercial citrus pectin sample and the samples of pectins isolated from *Z. marina* and *P. iwatensis *was highest at pH 6.0 and gradually lowered approximately by 10% and 50% with the decrease of pH to 4.0 and 3.0 respectively. Commercial pectin did not interact with the cerium ions at pH 2.0, 3.0 and 4.0, whereas pectin from *Z. marina* was slightly effective at pH 2.0. Only pectin from *P. iwatensis* effectively bound cerium ions at any pH used in the experiments (Figure 5).

Therefore, pectins isolated from seagrasses *Z. marina *and *P. iwatensis *have significantly higher cerium binding capacity in comparison with commercial pectin. This was proven by values of the maximum sorption capacity and affinity constant. Presumably it was determined by the lower degree of esterification as well as by the presence of side chains in the main homogalacturonan pattern.

The cation binding capacity of pectins is determined by the amount of non-methyl esterified galacturonosyl residues. Non-esterified pectins form gels in the presence of cations due to the formation of ionic cross-links between homogalacturonan chains. According to the “egg-box” model [22] cross-linked junction zones are formed between homogalacturonan chains containing at least six contiguous residues. Therefore, reduction of the degree of esterification causes dramatic increase of the metal binding capacity of pectins.

Cerium toxicokinetics mainly depends on the route of administration. It has been shown previously that orally administered cerium is selectively concentrated and precipitated as an insoluble form in enterocytes of the proximal intestinal tract after oral administration [23], suggesting that this mechanism of local concentration blocks dissemination of this element through the digestive barrier. Also small concentrations of cerium were found in inner organs with high blood flow such as liver, kidney, lungs and heart [24] indicating absorption of cerium from intestine into blood. Approximately 10% of absorbed cerium is excreted through feces and urine with retention of 45% in the liver, 35% in the skeleton, and 10% in other organs (primarily, the spleen and kidneys). In the blood cerium, either complexes with proteins or forms phosphate, hydroxide, and carbonate compounds in colloids. If the binding capacity of the protein transport system is not overwhelmed, cerium is transported to the liver and bones. If the system is overwhelmed, such as with i.p. dosing, colloidal aggregates are formed at the delivery site and then distributed to the liver and spleen [25].

Pectins as well as other dietary fibers with high degree polymerization are neither digested nor absorbed in the small intestine [26]. Therefore, they exert their binding properties in intestinal lumen. As the major part of cerium ions are concentrated in enterocytes of the proximal intestine [23], the pectin molecules can easily interact with the metal. In our previous research, the pectins were shown to alter behavior of lead in rats reducing lead storage in inner organs and bones after oral administration [27]. Mechanism of these effects is probably caused by misbalance of the lead concentrations in intestine and blood. These findings suggest that pectins may effectively remove cerium from intestine as well as from inner organs and even bones.

The results of the present study showed that pectins of marine origin exert more pronounced cerium binding effects than citrus pectin. This may be explained by the differences in their structure. Pectin from citrus, apple and other ground plants is considered to consist of a diverse set of structural elements, such as homogalacturonan (HG), xylogalacturonan (XGA), apiogalacturonan (AGA), rhamnogalacturonan I (RG-I) and rhamnogalacturonan II (RG-II). HG is mainly composed of partly methyl esterified stretches of α-1,4-linked-D*-*galacturonic acid (GalA) and has been suggested to consist of unique repeats of 30–200 α-D-GalA units [28]. Neighboring HG are linked together via one or two α-L-rhamnopyranose residues (α-L-Rha*p*) attached to the main chain through 1,2-link. From 8 to 74% of the carboxyl groups of α-D-GalA may be esterified with methanol. Usually the degree of esterification of citrus pectin varies from 60 to 70%.

Pectin contained in seagrasses typically possesses apiogalacturonan fragments where single or 1,3-linked D-apiose residues are attached to the D-galacturonic acid via 1,2- or 1,3-links [11]. RG-II makes up to 10–11% of the pectin and is generally composed of the homogalacturonan pattern with four side chains with quite a complicated structure. Also its contents may include 12 various types of sugars including unusual ones such as 2-*O*-methylxylose, 2-*O*-methylfucose, apiose and others [7]. Degree of esterification of the pectin from seagrasses is several folds lower than that of citrus pectin and this may dramatically affect its metal binding activity.

On the basis of these results we may conclude that the seagrasses studied in the experiments may be considered as a prospective source for manufacture of pectin containing pharmaceuticals purposed for binding and effective removal of radioisotopes including cerium from humans.

## 3. Experimental Section

### 3.1. Materials

Seagrass species *Z. marina *and *P. iwatensis *were gathered in Peter the Great Bay (the Sea of Japan) in September–October 2009. Pectins were isolated using the method of sequence acid hydrolysis with following extraction with sodium oxalate. For purification of extracted pectins they were dissolved in distilled water to make the 0.5% solution. Then three-fold volume of the 95% ethanol solution was used to precipitate pectin substances. Pectin gel was then separated by filtration with the consequent rinsing with 95% ethanol solution. The final pectin precipitate was rinsed and dried. This method is specific way to obtain the pure pectin substances containing no impurities [29]. Then the pectin samples obtained were characterized regarding the contents of total and free anhydrogalacturonic acid, degree of esterification and intrinsic viscosity of the pectin solutions. These pectin samples contained no microbial impurities because of the high ethanol concentrations used. As the pectin samples were purposed to be estimated regarding their metal-binding activity the amount metal ions in them was also assessed using chemoindicators. The analysis showed no metal ions contained by the pectin samples in the concentration enough to change the color of indicator.

Commercial high esterified citrus pectin without additives was obtained from Copenhagen Pectin A/S, Lille Skensved, Denmark. The stated degree of esterification of this preparation was 60.0%. The pectin preparation contained no acetyl or amide groups. All other chemicals were of the highest quality available. Distilled water was used throughout.

### 3.2. Pectin Analysis

The galacturonan content of the pectin preparation was determined colorimetrically by the m-hydroxydiphenyl method [30]. The degree of esterification was characterized using titrimetric analysis with 1 M NaOH solution in 50% ethanol in the presence of Hintone indicator [31]. Intrinsic viscosity of LMP was determined in 0.05 M NaCl/0.005 M Na-oxalate at 25.0 °C and pH 6.0 using an Ubbelohde viscosimeter. The intrinsic viscosity was related empirically to the molecular weight by the Mark–Howink equation [32] presented as µ =KM^α^ that is generally used for definition of the pectin molecular weight using values for the constants α (0.79) and K (216 × 10^−6^) that are suitable for pectins [33,34].

### 3.3. Experimental Procedures

0.1 M stock solutions (14.01 g L^−1^) of Ce^3+^ ions was prepared using analytical-reagent grade Ce_2_(SO_4_)_3_. The stock solution was diluted to give standard solutions of appropriate concentrations with controlled pH at 6.0 achieved by addition of either 0.1 M HCl or 0.1 M NaOH. Batch sorption experiments were conducted in 20 mL beakers and equilibrated using a magnetic stirrer. Then 1.0 mL aliquots of these standard solutions were placed in 20 mL beakers with 10 mL of solution containing 0.05 g of dry pectin preparation. Then the total volume of the solution was made up to 20 mL by addition of distilled water. Removal of pectin compounds from Ce^3+^ solution was performed by the use of centrifugal force unit at 3000 rpm for 10–20 min with following filtration through a glass filter with a pore size 100–120 µm. Concentration of Ce^3+^ ions in the supernatant obtained was analyzed using atomic absorption spectrophotometry method. The effect of Ce^3+^ sorption was studied within a pH range 2.0–6.0. The pH of the initial solution was adjusted to the required pH value using either 0.1 M HCl or 0.1 M NaOH. Pectins were equilibrated at the particular pH for 120 min at 400 rpm and at initial Ce^3+^ concentration of 0.6 g L^−1^ using a bath controlled at 24 °C. Each experiment was triplicated under identical conditions. A negative control experiment with no pectin added was simultaneously carried out to ensure that the cerium removal was caused by the polysaccharide binding activity and not by a beaker or filter. The parameters obtained were subjected to a one-way analysis of variance using a software package SPSS (Statistical Package for Social Sciences) for Windows (version 11.0; SPSS Inc.: Chicago, IL, USA, 2001) with a confidence level of 95% (*P* < 0.05).

The effect of agitation period was also studied to determine the optimum condition for sorption of Ce^3+^ ions. For batch kinetic studies 10 mL of solution containing 0.05 g of dry pectin were equilibrated at optimum condition as mentioned earlier. The sorption system was placed in 20 mL beakers and stirred by a magnetic stirrer. At the present time intervals, the aqueous samples (5 cm^3^) were taken and the concentration of Ce^3+^ was assessed.

Sorption equilibrium studies were conducted at optimum condition using a contact time of 120 min at pH 6.0. pH value 6.0 was considered as most acceptable because all polysaccharides at this point possess highest binding activity and the pH control requires minimum amounts of HCl and NaOH. Bath controlled temperature was 24 °C. Isotherm studies were conducted with a constant pectin preparation amount (0.05 g) and varying initial concentration of Ce^3+^ in the range of 0.05–0.7 g L^−1^. Each experiment was at least duplicated under identical conditions.

The metal accumulation (q) was determined as follows:


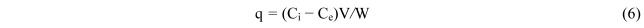


where C_i_ is the initial Ce^3+^ concentration (mg L^−1^), C_e_ is the final or equilibrium Ce^3+^ concentration (mg L^−1^), V is the volume of the Ce^3+^ solution (mL), and W is the weight of the dry samples of pectin (g).

The amount of the metal ions bound by the pectin compounds was expressed in mg g^−1^ of the dry pectin.

## 4. Conclusions

The source of pectin substances and their chemical structure substantially influence their metal binding activity. The main parameter affecting metal uptake of pectins is the amount of carboxyl group which are not occupied by methyl radical, *i.e.*, degree of esterification. The lower the degree of esterification, the larger is the amount of metal that can be bound by pectin molecules. Therefore, commercially high esterified pectins barely bind metal ions.

The pH change in the solution under similarconditions affects the dynamics of the binding process. Binding of the cerium ions by pectins is lower under low pH values, which is confirmed by values of Langmuir isotherm constants. The Langmuir sorption model is the best fit for describing the interaction between pectins and cerium ions, indicating that each active site of the polysaccharide binds a single metal ion. The rate of the process provided the equilibrium concentration in 60 min. 

Pectins isolated from *Phyllospadix iwatensis *exerts significantly more cerium binding in comparison with other pectins studied using any pH values and may be considered as a prospective source for development of new pharmaceuticals for removal of radioisotopes from the human body.
